# Radiographic characterization of the hands in Ritscher-Schinzel/3-C syndrome

**DOI:** 10.1186/2193-1801-2-594

**Published:** 2013-11-07

**Authors:** Kaitlyn J Friesen, Bernard N Chodirker, Albert E Chudley, Martin H Reed, Alison M Elliott

**Affiliations:** Department of Biochemistry and Medical Genetics, University of Manitoba, Winnipeg, Manitoba R3E 0W3 Canada; Department of Pediatrics and Child Health, University of Manitoba, Winnipeg, Manitoba R3A 1S1 Canada; Winnipeg Regional Health Authority, Program of Genetics and Metabolism, Health Sciences Centre, Winnipeg, Manitoba R3A 1R9 Canada; Department of Diagnostic Imaging, University of Manitoba, Winnipeg, Manitoba R3T 2N2 Canada

**Keywords:** Ritscher-Schinzel syndrome, 3-C syndrome, Metacarpophalangeal pattern (MCPP), Profile, Carpal height, Phenotype

## Abstract

**Abstract:**

Ritscher-Schinzel Syndrome (RSS) is a clinically variable, autosomal recessive disorder, involving cardiac, cerebellar and craniofacial abnormalities. Numerous reports describe hand changes in RSS patients; however, a detailed characterization of the hands has not previously been performed.

**Objective:**

The purpose of this study was to identify whether specific radiographic hand changes were characteristic of RSS and could serve as a diagnostic tool.

**Materials and methods:**

We performed a detailed radiographic hand characterization of 8 RSS patients. The patient population consisted of 5 males and 3 females from ages one month to 26 years, 7 months. The hands were characterized using metacarpophalangeal pattern (MCPP) profiles, carpal height and bone age analyses and assessment of bone morphology.

**Results:**

There was generalized brachydactyly with the second ray being the most severely affected. There was significant shortening of the first metacarpal and the fifth distal phalanx. The MCPP profile generated showed a consistent wavy pattern with average Z-scores ranging from -0.15 (4^th^ proximal phalanx) to -2.13 (1^st^ metacarpal) and 0.53 (4^th^ middle phalanx) to -1.73 (2^nd^ proximal phalanx) for the left and right hands, respectively. Six of eight patients showed a decreased carpal height. Bone age was within normal limits for all patients. Our study population showed consistent radiographic changes including: overtubulation of the bones (especially metacarpals 2-4), prominent tufts of the distal phalanges and a hypoplastic fifth distal phalanx.

**Conclusion:**

The hand findings identified in this study can provide helpful diagnostic tools to clinicians when the diagnosis of RSS is being considered.

## Introduction

Ritscher-Schinzel Syndrome (RSS) is a rare, autosomal recessive disorder first described by Ritscher et al. (Ritscher et al. 1987) who reported two sisters with similar craniofacial, cerebellar, and cardiac abnormalities. It is also commonly referred to as “3C syndrome” and is clinically heterogeneous (Leonardi et al. 2001). We have been following a large cohort of RSS patients who are First Nations from Northern Manitoba (Marles et al. 1995;Rusnak et al. 2008;Elliott et al. 2013). Seven of the eight patients included in this study have been previously reported (Marles et al. 1995;Elliott et al. 2013).

In addition to the craniofacial, cardiac, and cerebellar defects, numerous reports have discussed abnormalities involving the hands. Reported findings include: brachydactyly, camptodactyly, clinodactyly, syndactyly, single transverse creases, bilaterally adducted thumbs, absent flexion creases, proximally inserted thumbs, cutaneous syndactyly, and hypoplastic fingernails (Marles et al. 1995;Rusnak et al. 2008;Kosaki et al. 1997;Wheeler et al. 1999;DeScipio et al. 2005;Seidahmed et al. 2011). Despite clinical reports of hand anomalies, a detailed radiographic characterization of hand findings in RSS patients has not previously been performed.

Metacarpophalangeal pattern (MCPP) profiles have been used to aid in the diagnosis of congenital malformations and bone dysplasias since 1972. MCPP profiles are available for Turner Syndrome, hypochondroplasia, dyschondrosteosis, Marfan syndrome, and numerous other genetic disorders (Dijkstra et al. 1994;Laurencikas et al. 2006). MCPP profile analysis offers an objective and statistical method of identifying characteristic patterns in bone lengths that are unique to different syndromes. This method allows the unbiased comparison of patients of different ages and genders. The MCPP profile is a characteristic and relatively constant feature which remains unchanged with increasing age and can enable early diagnosis when a distinct MCPP profile has been identified (Laurencikas et al. 2006). Each profile has the ability to identify a developmental pattern that could help guide further molecular genetic analysis. MCPP profile analysis can be particularly useful in genetic syndromes and skeletal dysplasias with subtle clinical and radiological abnormalities, such as RSS.

Carpal height is a radiographic ratio with clinical significance in many syndromes and has been used as a diagnostic tool as well as to evaluate the severity and progression of a disease, such as arthritis (Poznanski et al. 1978;Poznanski 1984;Wang et al. 2010).

The purpose of this study was to characterize radiographic hand changes in patients with RSS. The patient population included five males and three females from one month of age to 26 years, 7 months. The data generated from the MCPP profile, carpal height analysis, bone age analysis, and assessment of bone morphology have the potential to describe a phenotypic pattern characteristic of RSS that could contribute to the diagnostic process.

## Materials and methods

The study was approved by the Health Research Ethics Board at the University of Manitoba. Informed consent was obtained from the parent/legal guardian of each patient. All patients participating in the study were diagnosed with RSS by a clinical geneticist and were followed by the Winnipeg Regional Health Authority Program of Genetics and Metabolism. The clinical information for all eight patients is summarized in Table 1. The principal inclusion criterion for this study was the availability of posterior-anterior hand radiographs. Measurements were obtained to the nearest tenth of a millimetre using a vernier caliper. The measurements of the nineteen tubular bones were obtained as described (Garn et al. 1972). Following measurement of the 19 tubular bones of the hand, the length of each bone was normalized and expressed as a Z-score (Z = (bone length-reference length/standard deviation)). The American reference data was utilized for standard values, as standards for First Nations populations are not available (Garn et al. 1972). The Z-scores were used to generate a visual comparison of the eight patients using an XY scatter plot. A mean Z-score was calculated for each bone by averaging the Z-scores of the eight patients. A graph of the mean values was generated to view an overall trend. Analysis of the output data was performed.

**Table 1:** **Clinical findings of RSS patients**

Patient	RSSH01	RSSH02	RSSH03	RSSH04	RSSH05	RSSH06	RSSH07	RSSH08
	(Patient III)*	(Patient VI)*	(Patient VIII)*					
**Gender**	F	M	F	M	M	M	F	M
**Age at time of radiography**	15 yrs 3 months	21 yrs 11 months	26 yrs 7 months	24 yrs 1 month	7 yrs 7 months	13 yrs 6 months	1 yr 8 months	1 month
**Craniofacies**								
Macrocephaly	+	+	+	+	+	-	-	+
Prominent Forehead	+	+	+	+	+	+	+	+
Brachycephaly	+	+	+	+	+	+	+	+
Low posterior hairline	+	+	+	+	+	+	+	+
Wide palpebral fissures	+	+	+	+	+	+	+	+
Hypertelorism	+	+	+	+	+	+	+	+
Coloboma	+	-	-	ND	-	ND	-	ND
Low set ears	+	+	+	+	+	+	Not documented	+
**CNS Finding**	Cranial ultrasound-no abnormality detected	Dandy-Walker cyst with hypoplasia of the vermis, abnormal gyri of cerebral cortex	Extra-axial fluid over cerebral hemispheres	Not imaged	Dandy-Walker variant with cerebellar vermis hypoplasia, hydrocephaly	Third, fourth and lateral ventricles prominent. Mild amount of extra-axial fluid within both frontal regions	Heterotopic grey matter adjacent to the occipital horn of both the left and right ventricles	Hypoplasia of the cerebellar vermis with associated dilatation of the 4^th^ ventricle, consistent with a Dandy-Walker variant
Intellectual Disability	+	+	+	+	+	+	+	+
**Cardiac Finding**	ASD/VSD, aberrant right subclavial artery, left sided superior vena cava joined at the coronary sinus	Muscular VSD with right ventricular hypertrophy	-	Limited study. No clinical evidence of cardiac disease.	ASD,VSD	Biventricular hypertrophy, intra-arterial defect	Large perimem- branous VSD + small PDA	-
Brachydactyly	+	+	+	+	-	+	+	+

*Marles et al. (1995) Am J Med Genet 56:343-350. ND = not documented, ASD = atrial septal defect, VSD = ventricular septal defect, PDA = patent ductus arteriosus. Patients RSSH02-06 and RSSH08 were included in the molecular analysis (Elliott et al., 2013).

RSSH07 (a 20 month old female) was compared to a 24 month old standard measurement due to the lack of data available for her age. The distal and middle phalanges of the fifth ray on the right hand of RSSH08 could not be measured due to positioning of the hand when the x-ray was taken.

Carpal height ratio was calculated as described (Poznanski et al. 1978;Keats et al. 1985) and was assessed by determining a standard deviation from the expected ratio between the second metacarpal (MC2) and the carpal height for a patient’s age and gender. The general morphology of the bones was also documented. The bone age was assessed by comparing the patient’s radiographs to standard radiographs for the corresponding age and gender of the patient (Greulich & Pyle 1959). These analyses were performed with a paediatric radiologist.

## Results

### Clinical findings

Table 1 reflects the variability in RSS. The craniofacial phenotype is consistent and distinctive in our patient population (Elliott et al. 2013). All of our patients have intellectual disability. There is variability with respect to the cardiac and cerebellar involvement.

### MCPP profiles

**Figure 1:**
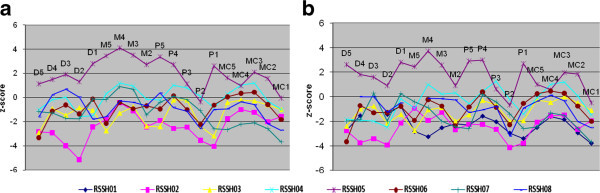
**MCPP profiles of the left (a) and right (b) hands of RSS patients.** Distal phalanges (D), Middle phalanges (M), Proximal phalanges (P) and Metacarpals (MC).

The MCPP profiles of the left and right hands are represented in Figure 1a, b respectively.

### Average MCPP profiles

**Figure 2:**
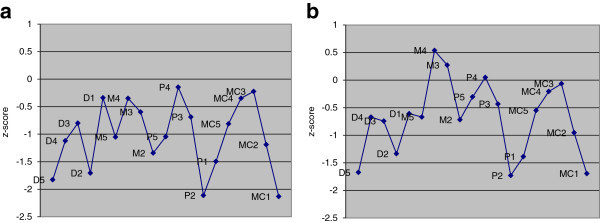
**Mean MCPP profiles on RSS patients. a** represents the mean of the left hands (8 patients), **b** represents the mean of the right hands (7 patients).

The mean MCPP profiles of the left and right hands are represented in Figure 2a, b respectively. The mean Z-scores are a result of averaging the Z-scores of eight patients for the left hand MCPP profile and seven patients for the right hand MCPP profile. The average Z-scores of the left hands ranged from a minimum -2.13 (1st metacarpal (MC1)) to maximum of -0.15 (4th proximal phalanx (PP)). The average Z-scores of the right hand were higher than those of the left hand with a minimum of -1.73 (PP2) and a maximum of 0.53 (4th middle phalanx (MP4)). Both graphs indicate brachydactyly with a significant shortening of the second ray. There is also notable shortening of the bones in the first ray. The Z-score of the fifth distal phalanx (DP5) and analysis of patient radiographs both indicate a significant abnormality. DP5 has one of the lowest Z-scores in both graphs and radiographic interpretation revealed it to be hypoplastic.

### Carpal height ratio and bone age

**Figure 3:**
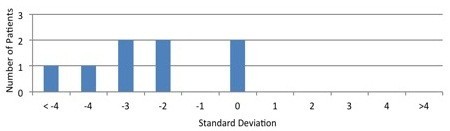
**Carpal height standard deviations of RSS patients.**

Figure 3 displays the standard deviations of the carpal height ratio in the patient population. Six out of eight patients were below average (between -2 and -4 standard deviations). Two patients had an average carpal height.

The bone ages were not significantly different from normal for all patients.

### General morphology of hands

**Figure 4:**
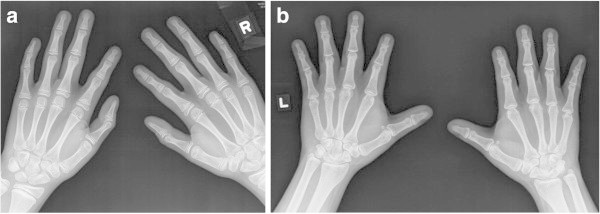
**Bilateral hand X-ray of a 13 year old male (a) and a 22 year old male (b) with Ritscher-Schinzel Syndrome. a** The fifth distal phalanges are hypoplastic, the bones are overtubulated (especially metacarpals 2-4) and the distal phalanges have prominent tufts. **b** Metacarpals 2-4 are overtubulated, the distal phalanges short, and the fifth distal phalanges are gracile.

Phenotypic analysis of the radiographs indicated similar findings including: bilateral prominent tufts of the DPs (5/8 patients), bilateral overtubulation of bones (especially MCs 2-4) (6/8 patients) and bilateral hypoplastic fifth DPs (5/8 patients) (Figure 4a and b).

## Discussion

### Clinical findings

Not all three systems are involved in all RSS patients (Leonardi et al. 2001;Elliott et al. 2013). An explanation for the diversity of cardiac malformations as evidenced in Table 1 has been proposed as a “shift” of the threshold (Lurie & Ferencz 1996). Six of the patients in Table 1 (RSSH02-06 and RSSH08) were included in a molecular study and were homozygous for the same mutation in the *KIAA0196* gene, which encodes a highly conserved protein, strumpellin (Elliott et al. 2013).

### MCPP profile of RSS patients and comparison

The MCPP profile revealed a distinctive wavy pattern (Figure 1a, b). In general, the average MCPP profile demonstrated brachydactyly with significant shortening in particular bones. The first MC, the second PP, and the fifth DP were the most shortened. The tubular bones of the second ray, also affected, resulting in a shortened index finger and can be a helpful clinical sign. The bones of the first ray become less affected from MC to DP, while the bones of the fifth ray become more affected from MC to DP. The DPs of the third and fourth rays were also affected. RSSH05 was a significant outlier due to increased values however; the overall pattern was similar. All growth parameters (height, weight, head circumference) for this patient were increased. Although he lacked ocular coloboma, he showed the craniofacial features typical of RSS, intellectual disability, Dandy-Walker variant with cerebellar vermis hypoplasia, atrial septal defect and ventricular septal defect (Table 1). Furthermore, this patient was confirmed to have the same molecular defect (Elliott et al. 2013).

The pattern displayed by our patient population was distinct from that of other genetic syndromes such as Turner Syndrome, which also displays syndromic brachydactyly but demonstrates a bone-shortening gradient with increasing shortening from DPs to MCs in all rays (Laurencikas et al. 2005). Leri-Weill Dyschondrosteosis and Noonan Syndrome also show syndromic brachydactyly however each displays characteristic patterns (Butler et al. 2000;Laurencikas et al. 2005).

The patients in this study demonstrated brachydactyly. Brachydactyly can be isolated or syndromic. The isolated brachydactylies have been characterized into five groups, A-E, including several subgroups (Mundlos 2009). Analysis of different types of brachydactyly can provide insight into potential developmental pathways disrupted in RSS. Although all tubular bones are affected in RSS patients, the second ray is the most significantly affected followed by the first and fifth rays. Brachydactyly B2 exhibits some characteristics displayed by our RSS patient population including: hypoplasia/aplasia of the distal phalanges and proximally inserted thumbs as a result of a shortened first metacarpal and is caused by a missense mutation in Noggin (NOG), which under normal circumstances forms a homodimer which binds to bone morphogenic proteins (BMP). Like RSS, Brachydactyly E demonstrates a shortening of all the metacarpals. Overall, the pattern observed in the RSS population is distinctive from the isolated or syndromic brachydactylies. The osseous hand and skull involvement in RSS patients suggests a potential role of the strumpellin protein in chondrogenesis.

### Carpal height ratio analysis

The average standard deviation of our RSS patients was negative indicating a decrease in carpal height. In our study, the second metacarpal (MC2) was shortened. This bone is utilized when calculating the ratio. A shortened MC2 would have the tendency to skew the result toward a greater carpal height (increased standard deviation). This was not the case for our patient cohort. Decreased carpal height and other carpal abnormalities have been found in other genetic syndromes such as Poland Syndrome (Friedman et al. 2009). The overall morphology of the carpals was normal despite the decrease in carpal height. Although this finding does not contribute to further understanding of the pathogenesis of the disorder, it can be utilized as a phenotypic marker for RSS.

### Bone Age and radiographic features

The bone ages of the study population were not significantly different from expected. Zankl et al. performed a follow up study of the original patients (Zankl et al., 2003). One of the sisters appeared to have a delayed bone age. Our RSS study population showed normal bone ages, indicating that this is not a consistent feature in all RSS patients.

The observed radiographic features of our RSS patients included: bilateral prominent tufts of the DPs, bilateral overtubulation of bones (especially MCs 2-4), and bilateral hypoplastic fifth DPs.

## Conclusion

The wavy MCPP profile, with a shortened second ray, MC1 and DP5, decreased carpal height and other radiographic features identified in this study offer new diagnostic tools for clinicians when a diagnosis of RSS is being considered.
